# A fully electronic intensity-modulated radiation therapy quality assurance (IMRT QA) process implemented in a network comprised of independent treatment planning, record and verify, and delivery systems

**DOI:** 10.2478/v10019-010-0017-9

**Published:** 2010-05-24

**Authors:** Daniel W Bailey, Lalith Kumaraswamy, Matthew B Podgorsak

**Affiliations:** 1 Department of Physics, State University of New York at Buffalo, Buffalo NY, USA; 2 Department of Radiation Medicine, Roswell Park Cancer Institute, Buffalo NY, USA

**Keywords:** EPID, IMRT, QA, paperless, portal dosimetry, PACS: 87.53.Bn, 87.53.Kn

## Abstract

**Background:**

The purpose of this study is to implement an electronic method to perform and analyze intensity-modulated radiation therapy quality assurance (IMRT QA) using an aSi megavoltage electronic portal imaging device in a network comprised of independent treatment planning, record and verify (R&V), and delivery systems.

**Methods:**

A verification plan was generated in the treatment planning system using the actual treatment plan of a patient. After exporting the treatment fields to the R&V system, the fields were delivered in QA mode with the aSi imager deployed. The resulting dosimetric images are automatically stored in a DICOM-RT format in the delivery system treatment console computer. The relative dose density images are subsequently pushed to the R&V system. The absolute dose images are then transferred electronically from the treatment console computer to the treatment planning system and imported into the verification plan in the dosimetry work space for further analysis. Screen shots of the gamma evaluation and isodose comparison are imported into the R&V system as an electronic file (e.g. PDF) to be reviewed prior to initiation of patient treatment. A relative dose image predicted by the treatment planning system can also be sent to the R&V system to be compared with the relative dose density image measured with the aSi imager.

**Results:**

Our department does not have integrated planning, R&V, and delivery systems. In spite of this, we are able to fully implement a paperless and filmless IMRT QA process, allowing subsequent analysis and approval to be more efficient, while the QA document is directly attached to its specific patient chart in the R&V system in electronic form. The calculated and measured relative dose images can be compared electronically within the R&V system to analyze the density differences and ensure proper dose delivery to patients.

**Conclusions:**

In the absence of an integrated planning, verifying, and delivery system, we have shown that it is nevertheless possible to develop a completely electronic IMRT QA process.

## Introduction

Intensity-modulated radiation therapy (IMRT) involves complex treatment plans that are completely patient specific in order to highly conform delivered dose to the treatment volume, thus improving normal tissue sparing as compared to more traditional radiotherapy techniques.^1^,^2^ As a consequence, the complexity and uniqueness of these treatment plans demand patient-specific pretreatment quality assurance (QA) of all IMRT treatments. Standard methods of IMRT QA involve ionization chambers, diode arrays and radiographic films, often used in some combination to provide verification of absolute dose, field geometry, number of monitor units, etc. However, these traditional QA methods carry some distinct disadvantages, especially in the clinic that delivers a large number of IMRT treatments. These methods can be exceedingly time and resource demanding, requiring calibration and constancy checks of ionization chambers, set-up and calibration of diode arrays, calibration of films and expensive processing (unless self-developing dosimetry film is chosen as a more convenient yet still expensive alternative).

Furthermore, for all of the QA methods listed above, the QA analysis report may not be readily available in electronic form, demanding direct attachment to the patient paper chart (or manual scanning into the patient electronic chart, a process that is still not paperless). The disadvantages of paper charts are well known and well documented^3^ - including illegible signatures, notes and prescriptions; inaccessibility to multiple reviewers at one time; difficulties in locating charts and inability to access them remotely; etc. Meanwhile, the benefits of implementing an entirely paperless electronic medical record process have also been expounded in the literature.^3^–^7^ A study published by the National Institute of Health and the Journal of the American Medical Association concluded that “EMRs will eventually become the standard of care,” citing that electronic patient charts provide complete, legible, and organized patient information in a format that is accessible at any time, even to multiple viewers in multiple (even remote) locations.^3^ Given the current shift toward adopting electronic medical records over paper charts, it is all the more important that the pretreatment IMRT QA process be fully electronic: no films, no printing and no scanning of QA reports, treatment plans and other documents.

In recent years, it has been demonstrated that an electronic portal imaging device (EPID), previously employed to replace radiographic portal images for patient alignment, can effectively be used for absolute dose measurement and pretreatment IMRT verification.^8^–^12^ In EPID IMRT QA, portal dosimetric images are compared to respective portal dose predictions created by a treatment planning system (TPS) using geometric and dosimetric tools (such as dose profiles and gamma evaluation).^13^–^15^ Thus, with a properly calibrated and commissioned EPID, all qualitative and quantitative data necessary for verification of an IMRT fluence is acquired in a single exposure, and all information is readily available in electronic form allowing for the possibility of an entirely paperless IMRT QA process.

A significant roadblock to the paperless EPID IMRT QA process is the common situation in which the treatment planning, record and verify (R&V), and radiotherapy delivery systems are not manufactured by the same vendor and thus communication between these systems is not entirely integrated. The purpose of this study is to implement a fully electronic method to perform and analyze patient-specific IMRT QA using an EPID in a network comprised of independent treatment planning, R&V, and delivery systems. The advantages of such a QA process over standard methods of IMRT QA include:

1. Excellent efficiency, acquiring complete qualitative and quantitative information in a single exposure for each field, with no processing and no other calibration than the absolute and relative dose calibrations of the EPID (at intervals suggested by the vendor).
2. Excellent resolution compared to ionization chambers and diode arrays, with arrays as high as 1024x768 pixels with 0.392 mm pixel pitch (Varian PortalVision aS1000, Varian Medical Systems, Palo Alto CA).
3. Possibility of weekly QA by quick acquisition of EPID relative dose density images and comparison within the R&V system to TPS predictions of those dose densities.
4. IMRT QA report electronically attached to the patient chart within the R&V system in a paperless process with no manual attachment or tracking of QA reports, thereby decreasing the probability of errors (e.g. misplacement of QA document, etc.).

## Methods

We have commissioned an electronic portal dosimetry system consisting of an amorphous silicon (aSi) EPID (Varian PortalVision aS1000), coupled to a Varian Trilogy linear accelerator with the Varian Millinium Multi-Leaf Collimator (MLC, 120 leaves). The PortalVision aS1000 is a 40x30 cm^2^ flat-panel, indirect detection EPID with a matrix of 1024x768 pixels with 0.392 mm pixel pitch. For this study, all EPID images were acquired at the minimum SSD of 105 cm with gantry and collimator at zero degrees (unless the collimator needed alternate positioning to avoid regions of high backscatter in the EPID).^16^ The EPID was fully calibrated using the procedures supplied by the vendor^17^, using the following intervals: the dark field background correction and flood field relative dose calibration were both performed weekly; while the absolute dose calibration was performed each day that the EPID was in use for IMRT QA (also employing the diagonal dose profile correction suggested by Bailey *et al*.^16^). The beam symmetry, energy and output were verified each week. The TPS employed for this study is Varian Eclipse (Version 8.6, including Portal Dosimetry Version 8.2.24), and the R&V system is the vendor-independent Impac Mosaiq (Version 1.6, Elekta Oncology Systems, Norcross GA).

### From TPS to the R&V system

Our electronic QA process begins with a patient-specific radiotherapy treatment plan created in the TPS using inverse-planning IMRT techniques based upon the patient’s 3-D computed-tomography (CT) data and the dose criteria predefined by the radiation oncologist. Each specific treatment field within this plan contains 320 control points that dictate the dynamic motion of the MLC leaves. Firstly in this process, the TPS uses the input geometric and dosimetric criteria to calculate an ideal fluence matrix referred to as the optimal fluence. Secondly, the optimal fluence is sent to the Leaf Motion Calculator which incorporates various mechanical and geometric aspects of the delivery system (*e.g.* MLC beam transmission, minimum leaf gap, maximum leaf speed, MLC position deviation tolerance, etc.) to calculate leaf trajectories for the fluence that the system can capably deliver, known as the actual fluence.^18^ Routine IMRT QA is partly designed to check the accuracy of these beam models and parameters. If the necessary LINAC collimator jaw settings are beyond a certain separation (approximately 15 cm), the TPS splits the treatment field into multiple overlapping carriages, maintaining maximum degrees of freedom in MLC position and motion. After the treatment plan is completed and approved, the plan is electronically exported to the R&V system as a DICOM-RT file which includes all necessary patient information and delivery information, such as number of monitor units (MU), dose rate, collimator settings, and dynamic MLC positions.

### From R&V system to LINAC delivery

The R&V system communicates the delivery parameters from the TPS to the delivery system (and allows for automatic field setup), and further provides an electronic medical record (EMR) which tracks the fractions and doses that have been delivered to the patient, the delivery system settings for each field and fraction delivered, portal images and IMRT QA dosimetric images acquired with the EPID (or scanned films), among other information. When the treatment plan is delivered, whether for pretreatment QA or actual treatment delivery, the R&V system communicates the field setup and delivery information to the LINAC delivery system as an RTP file and stands by to record the subsequent delivered parameters and capture the acquired images. The IMRT QA process also checks the accuracy of communication and file transfer between the R&V and delivery systems for each delivered field.

### From image acquisition to the electronic medical record

**FIGURE 1:**
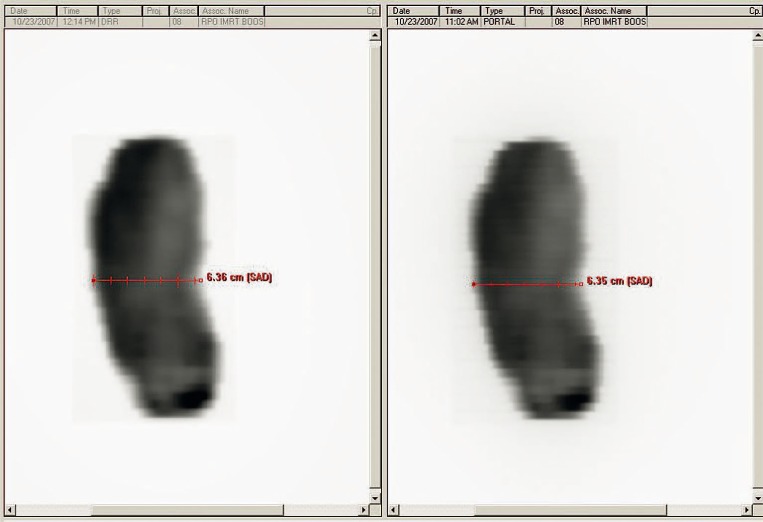
2D integrated relative dose images displayed in the R&V software: 1) acquired using the EPID in integrated acquisition mode (right); and 2) predicted by and exported from the TPS (left). These images are saved within the patient’s EMR, attached directly to the appropriate treatment field, and can be compared with various measuring tools within the R&V software (for example, the measuring tool illustrated in the figure).

**FIGURE 2:**
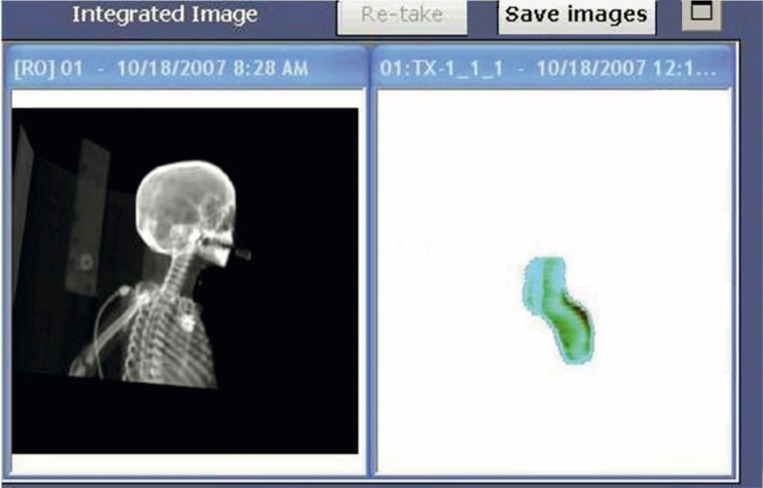
Absolute dose image computed from the EPID response and the most recent calibration data for the appropriate energy and dose rate, as displayed by the delivery system computer upon acquisition. This image is exported from the delivery system treatment console computer to the TPS for comparison to the calculated portal dose prediction for the appropriate field and SSD.

In order to acquire IMRT dosimetric images with the Varian delivery system and portal imager, the EPID is positioned with the center of the detecting surface aligned to the LINAC cross-hairs and at the desired SSD (minimum of 105 cm, maximum of 140 cm). Since the Varian TPS is programmed to predict non-transit EPID response, no phantom or other buildup is placed between the source and the EPID detecting surface. The delivery system is prompted by the user to acquire a portal dose image for each field, and the image must be acquired in “Integrated Acquisition” mode, meaning that the EPID continuously collects data throughout the duration of beam-on time (with maximum readout of 20–30 frames per second^19^) with no dependence on the timing of LINAC beam pulses, and sums all the collected data from one acquisition to form one image. The patient plan is delivered from the R&V system in QA Mode such that the delivery does not contribute to the tracking of patient dose delivery, but the chart reviewer can see whether or not the fields have been delivered for QA. When the delivery of a single field is complete, the delivery system calculates two images simultaneously from one acquisition: (1) an integrated relative dose image of the fluence (Figure 1, right panel), and (2) an absolute dose image computed from the EPID response and the most recent calibration data for the appropriate energy and dose rate (Figure 2). The absolute dose image can be collected from the delivery system treatment console computer via portable drive or network (we have used both methods), but cannot be automatically exported to the R&V system since this system has no information about the dosimetric calibration of the imager. However, a filter can be set up within the R&V system to automatically collect the relative dose density image and attach it to the respective field within the patient’s EMR (in DICOM-RT format).

## Results and discussion

Analysis of the acquired EPID images takes two paths, one for the relative dose density image and one for the absolute dose image.

### Qualitative analysis

As mentioned in the previous section, the relative dose density image is collected by the R&V system and attached to the specific patient field. To check the field geometry and relative dose distribution, a respective planned relative dose density map must be exported from the TPS (for the same SSD at which the EPID image was acquired) and similarly attached to the specific field. In this manner, the planned fluence and the acquired fluence can be placed side by side in the R&V software for qualitative comparison (Figure 1). This process is analogous to comparing a TPS printout of the fluence at a certain SSD to a radiograph exposed to the same IMRT field at the same SSD as the printout. The R&V software contains a number of measuring tools which can be used to compare the field size, leaf position, qualitative dose distribution, etc.

Though this type of QA does not contribute substantial amounts of information to the absolute dose QA (discussed below) when performed only once, it does have one distinct advantage. Currently, daily QA for IMRT treatments is virtually non-existent (though some institutions are pursuing *in vivo* QA with EPIDs).^20^–^22^ However, one of the main objectives of patient-specific QA is to ensure that the electronic files containing treatment and delivery system information accurately reflect what was planned and approved in the TPS. If the EPID were used to take a quick non-transit image of one or two fields in the radiotherapy plan (much the same way portal images are currently used for patient positioning), these EPID images can easily and quickly be compared to the fluences already exported from the TPS and stored in the patient’s EMR. In this way, radiotherapy professionals can quickly verify that, throughout the course of treatment and daily file transfer, the correct treatment fields and DMLC positions are being delivered accurately. Using this technique, it may also be possible to catch mechanical problems (such as errors in MLC leaf and collimator jaw positions) before the patient is treated, even between the extensive monthly LINAC QA intervals. Thus, this quick, qualitative analysis with the grayscale EPID image could be used on a weekly basis to provide fast and efficient system QA, much as weekly port films (with static MLC) are used to provide clinical treatment QA.

### Quantitative analysis

To complete quantitative analysis on the absolute dose EPID image, this file must first be exported from the delivery system treatment console computer via portable drive or network connection, and imported to the computer with which the analysis will be completed. It is possible to perform this analysis via custom made software^10^,^12^,^18^, commercially available software modalities alternative to the TPS in use^23^ (see EPIDose, SunNuclear, Melbourne, FL), or the portal dose prediction and analysis capabilities of the TPS in use. We currently employ the Varian Portal Dosimetry algorithm (Dosimetric Portal Image Calculation, DPIC) within the Eclipse TPS to create portal dose predictions for the aS1000 PortalVision EPID at desired SSD. Commissioning of this algorithm requires capturing two vendor-specified EPID images (at two different SSDs), the diagonal beam profile measured during LINAC commissioning (*i.e.* along the major diagonal of a 40 x 40 cm^2^ field of desired energy at d_max_ in water), and the EPID acquisition of field-size output factors for various field sizes specified by the vendor.^24^,^25^ To perform IMRT QA with the PortalVision EPID, the Varian TPS has been programmed to predict the response of the EPID to an IMRT field delivered with no buildup or phantom between the MLC and the EPID, following the methods pioneered by Van Esch *et al*. in 2004.^11^,^20^

**FIGURE 3:**
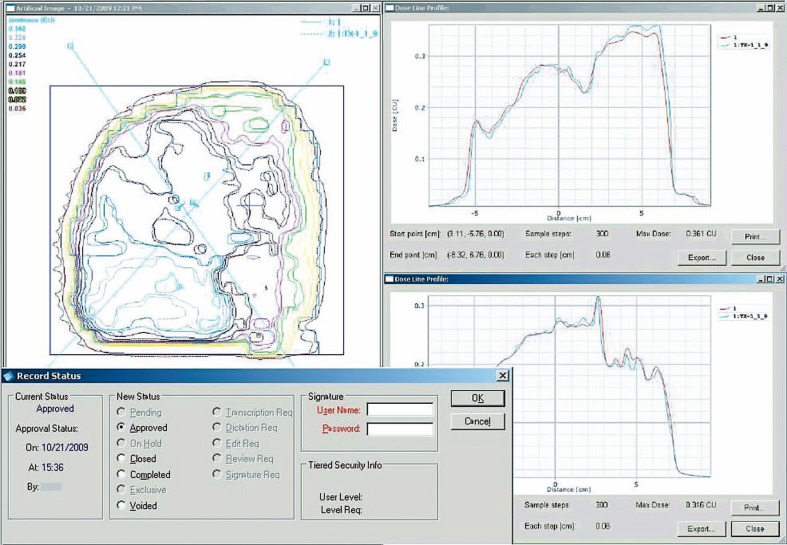
Portal dose prediction and acquired EPID absolute dose image as compared in the TPS via: 1) predicted vs. measured isodose lines (left panel); and 2) predicted vs. measured dose line profiles (right two panels). This analysis (in PDF or other desired format) is attached to patient’s EMR in the R&V software for approval prior to treatment. The bottom left panel shows the record status dialogue window within the R&V system, including reviewer options such as “pending,” “approved,” “voided,” etc.

With the EPID dose image imported into the TPS and the respective portal dose prediction calculated, these two planar dose maps can be evaluated through dose difference analysis, gamma evaluation, dose profile line scans, isodose comparisons, various measuring tools, etc. (Figure 3). For gamma evaluation and dose evaluation, the region of interest can be selected to only include the area of the detector within the collimator jaws, or a low-dose threshold can be specified by the user which effectively limits the analysis to the image within the collimator jaws. The resolution of the EPID image and subsequent analysis is far superior to ionization chambers and 2D arrays, while the ease of calibration and image analysis is far more resource and time efficient than the use of films.

To complete the IMRT QA report, the QA analysis can be easily and electronically transferred from the TPS to the patient’s EMR in the R&V system by copying the screen to any standard word processing or image editing software, or the screen can similarly be printed to PDF or postscript with the appropriate open-source software installed. A QA report can thus be created for each field within the radiotherapy plan and electronically attached to the patient’s chart, requiring no paper, no films, no scanning documents, and no searching for misplaced QA reports. Furthermore, the R&V system can be set up such that this QA analysis must be approved before the fields are treated (see the status dialogue window in Figure 3).

## Conclusions

Our radiotherapy department does not have integrated planning, R&V, and delivery systems - and yet we have shown that even in this hybrid environment it is nonetheless possible to develop a completely electronic IMRT QA process. Given the current demand for paperless patient charts, developing a paperless IMRT QA process is vital, even in systems that understandably include components made by diverse vendors. The process suggested in this study is paperless, filmless, time saving and reliable, enabling the pretreatment IMRT QA process to be far more efficient. Furthermore, QA analysis documentation can be directly attached to its specific patient EMR within the R&V system, eliminating searching for documents and running around to obtain signatures, while greatly reducing the risk of misplacing or losing the QA report. The calculated and measured relative dose density images can be viewed electronically side by side within the patient’s EMR to quickly and qualitatively analyze the density differences, field sizes and MLC trajectories, ensuring proper dose delivery to patients - even on a weekly basis. The absolute dose EPID images can be analyzed quickly and thoroughly with custom software or programs supplied by the TPS vendor or a secondary vendor, providing an absolute dose verification system that is of substantially higher resolution than arrays of diodes or ionization chambers, and substantially more efficient than exposing, processing, calibrating, scanning, analyzing and storing films.
